# Gut dysbiosis in nonalcoholic fatty liver disease: pathogenesis, diagnosis, and therapeutic implications

**DOI:** 10.3389/fcimb.2022.997018

**Published:** 2022-11-08

**Authors:** Jie Fang, Chen-Huan Yu, Xue-Jian Li, Jin-Mei Yao, Zheng-Yu Fang, Soo-Hyun Yoon, Wen-Ying Yu

**Affiliations:** ^1^ Zhejiang Provincial Laboratory of Experimental Animal’s & Nonclinical Laboratory Studies, Hangzhou Medical College, Hangzhou, Zhejiang, China; ^2^ Institute of Cancer and Basic Medicine, Chinese Academy of Sciences, Hangzhou, Zhejiang, China; ^3^ Zhejiang Cancer Hospital, University of Chinese Academy of Sciences, Hangzhou, Zhejiang, China; ^4^ Department of Clinical Laboratory Medicine, The First Affiliated Hospital, Zhejiang University School of Medicine, Hangzhou, Zhejiang, China; ^5^ Institute of Medical Science, Wonkwang University, Iksan, South Korea

**Keywords:** gut dysbiosis, nonalcoholic fatty liver disease, bile acid metabolism, probiotics, novel treatment strategies

## Abstract

The incidence of nonalcoholic fatty liver disease (NAFLD) is increasing recently and has become one of the most common clinical liver diseases. Since the pathogenesis of NAFLD has not been completely elucidated, few effective therapeutic drugs are available. As the “second genome” of human body, gut microbiota plays an important role in the digestion, absorption and metabolism of food and drugs. Gut microbiota can act as an important driver to advance the occurrence and development of NAFLD, and to accelerate its progression to cirrhosis and hepatocellular carcinoma. Growing evidence has demonstrated that gut microbiota and its metabolites directly affect intestinal morphology and immune response, resulting in the abnormal activation of inflammation and intestinal endotoxemia; gut dysbiosis also causes dysfunction of gut-liver axis *via* alteration of bile acid metabolism pathway. Because of its composition diversity and disease-specific expression characteristics, gut microbiota holds strong promise as novel biomarkers and therapeutic targets for NAFLD. Intervening intestinal microbiota, such as antibiotic/probiotic treatment and fecal transplantation, has been a novel strategy for preventing and treating NAFLD. In this article, we have reviewed the emerging functions and association of gut bacterial components in different stages of NAFLD progression and discussed its potential implications in NAFLD diagnosis and therapy.

## Introduction

Nonalcoholic fatty liver disease (NAFLD) is the most common cause of chronic liver injury disease worldwide, with a broad spectrum ranging from simple steatosis, nonalcoholic steatohepatitis (NASH) to cirrhosis and even hepatocellular carcinoma (HCC). Epidemiological investigation shows that NAFLD has become one of the most common liver diseases, with a global prevalence of 25.2%, 27.4% in Asia (Lonardo et al., 2016; Wiest et al., 2017), but more than 33% in China (Zhou et al., 2020; Wong and Chan, 2021). As NAFLD is a “silent” disease, the actual number of people with this disease is likely to be higher. Recently, growing evidence displays that NAFLD is multisystem metabolic disease associated with high insulin resistance and genetic susceptibility, in which the accumulation of triglycerides in hepatocytes is the main pathological change (Brunt et al., 2015). Besides liver-related complications, NAFLD also increase the risk of type 2 diabetes mellitus (T2DM), cardiovascular and chronic kidney disease. Conversely, obesity, T2DM, lifestyle changes and druggable genetic alterations also rise the morbidity and mortality of NAFLD (Mohammadi et al., 2020). Despite advance in the pathogenesis of NAFLD, few therapeutic drugs are available. Although nonspecific antioxidative, anti-inflammatory, hypolipemic and hypoglycemic drugs have been widely used in clinic to improve the symptoms, knowledge on NAFLD pathogenesis remains incomplete and hence there are few FDA-approved medications that directly treat NAFLD. In addition, interventions for NAFLD have focused on diet and lifestyle changes, but outcomes are unsatisfactory due to poor patient compliance (Notarnicola et al., 2021). Due to the hidden lesions, 20% patients have an irreversible advanced fibrosis and even HCC before the initial diagnosis (Leung et al., 2022). Therefore, the early diagnosis is important for ensuring appropriate treatment to delay or control the NASH progression. Current clinical diagnosis of NAFLD involves various procedures such as blood tests, B-ultrasound, transient elastography and MRI, but misdiagnosis of nonspecific symptoms (HBV-associated hepatitis, alcoholic hepatitis, and cirrhosis) is difficult to be distinguished in clinical practice, indicating that effective and reproducible biomarkers are still needed in clinical application (Cusi et al., 2022).

The popular theory for the pathological progression of NAFLD is “multi-hit” theory (Ore and Akinloye, 2021). According to this theory, the vicious cycle of “lipid accumulation/steatosis → hepatic lipotoxicity → metabolic disorders → inflammatory response → insulin resistance → aggravation of metabolic disorders” forms in the liver through interactions among various factors. The dysbiosis of the gut microbiota can be involved in multiple attacks on the liver and plays a key role in the pathogenesis of NAFLD. The intestinal bacteria and their metabolites can enter into the liver through the portal vein and subsequently affect the pathophysiological processes of liver. The gut microbiota and its metabolites can act as the molecular vehicles between intestine and liver (Wiest et al., 2017; Nawrot et al., 2021). Amazingly, some specific gut microbiota and metabolites, including short-chain fatty acids (SCFAs), bile acids (BAs), lipopolysaccharide (LPS), choline and trimethylamine (TMA), would be changed associated with disease severity and fibrosis stage, indicating the potential of diagnostic markers for NAFLD (Del Chierico et al., 2017; Loomba et al., 2019; Lee et al., 2020). Several clinical trials involving gut microbiota intervention, including probiotic and prebiotic supplementation, fecal microbial transplantation (FMT), and microbiome-targeted therapies (MTT), are underway for nearly every type of liver disease (Behrouz et al., 2020; Liu et al., 2021). As reported, the overall survival rates of patients with severe liver diseases who received FMT from healthy donors could be increased by 54.2% compared with those without FMT, indicating that the gut microbiota directly affects NAFLD progression (Philips et al., 2017). Therefore, the regulation, modification, and reconstruction of the gut microbiota may become a personalized strategy for the prevention and treatment of NAFLD.

## Roles of the gut microbiota dysbiosis on NAFLD occurrence and development

**Figure 1:**
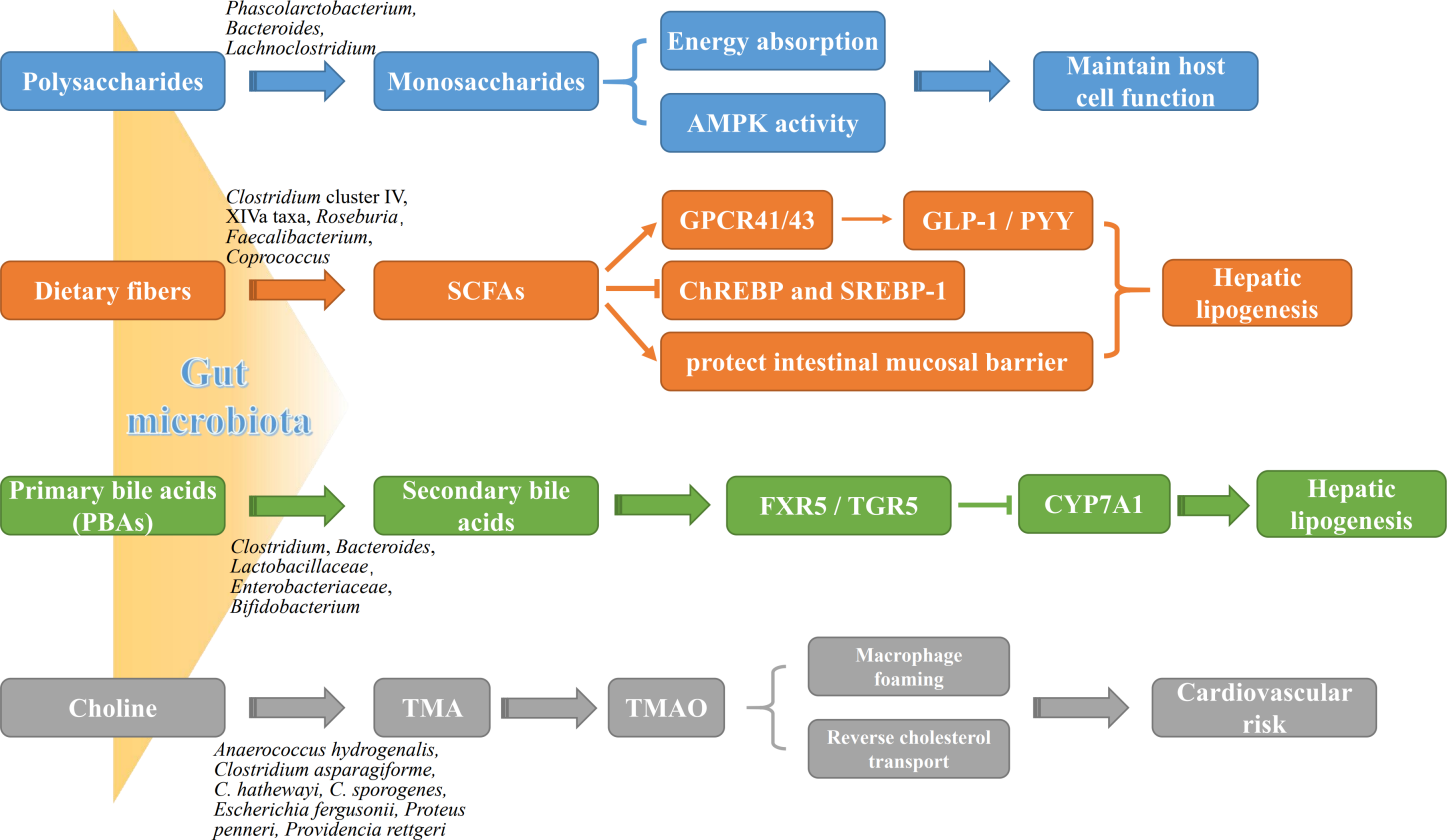
The roles of gut microbiota metabolites in NAFLD pathogenesis. Gut microbial metabolites, such as monosaccharides, SCFAs, BAs and TMA, not only are potently involved in energy metabolism of liver and intestinal epithelial cells, but affect directly liver lipogenesis and systemic inflammation. AMPK, adenosine monophosphate-dependent protein kinase; ChREBP, carbohydrate-responsive element-binding protein; CYP7A1, cytochrome P450 7A1; FXR, farnesoid X receptor; GLP-1, glucagon-like peptide-1; GPCR41/43, G protein-coupled receptor 41/43; PYY, peptide YY; SREBP-1, sterol regulatory element-binding protein 1; TGR5, takeda G protein receptor 5; TMAO, trimethylamine oxide.

The gut microbiota is a complex community that exist in the human gastrointestinal tract in a symbiotic manner and maintain 3 important physiological functions of the human digestive system, namely digestion, metabolism, and protection (Stražar et al., 2021; Zheng et al., 2021). Growing studies have demonstrated several mechanistic pathways of gut microbiota involved in the progression of NAFLD (**Figure 1**).

### Gut microbiota affects host energy metabolism by altering intestinal metabolites

The gut microbiota breaks down undigested polysaccharides into monosaccharides and dietary fibers into SCFAs by utilizing a variety of enzymes, both of which provides energy support for the host cells. Most notably, SCFAs exert multiple benefits in the regulation of metabolism and immune, and could be emerged as a potential therapeutic agent against various diseases. Beside the main source of energy for host colonocytes, SCFAs have distinctive pharmacological and physiological functions, such as promoting colonic motility, protecting intestinal mucosal barrier, altering carbohydrate and lipid metabolism, participating in immune regulation, and improving the absorption of electrolytes and nutrients as well as anti-inflammatory and antitumor activities (Rauf et al., 2022). Depending on the amount of dietary fiber intake, approximately 500-600 mmol of SCFAs are produced in the colon daily. Among all the metabolic products of carbohydrate fermentation by the microbiota, the acetic acid (C2), propionic acid (C3) and butyric acid (C4) are the most abundant SCFAs in the intestinal tract. Acetic acid is an important source of host energy, providing approximately 10% of total energy daily for the body. Butyric acid as the main source of energy for supplement epithelial cells plays a crucial role in the function of the intestinal barrier; and is the main metabolic substrate for the gastrointestinal microbiota, providing at least 60-70% of its energy requirements for their proliferation and differentiation (Zhou et al., 2018). It has been demonstrated that the intestinal epithelial cells both in germ-free mice and in antibiotic-induced microbiome depletion mice present shorter turnover and lower proliferative activity than the normal mice, and decreased availability of SCFAs shifts colonocyte energy metabolism toward glucose utilization (Slezak et al., 2014; Park et al., 2016). Moreover, butyric acid inhibited the activation of ChREBP and SREBP-1, resulting in the repressing of lipogenesis (Park et al., 2016). Propionic acid is mainly catabolized in the liver, where it involves in the transformation from pyruvate to glucose, and it also has been found to reduce lipid accumulation in overweight and adipose sufferers (Canfora et al., 2017). Unlike butyric acid and acetic acid acting as sources of energy for host cells, propionic acid is the precursor for adipogenesis and gluconeogenesis, which plays more important roles in the pathogenesis of NAFLD (Liu et al., 2021). Interestingly, no matter what type of dietary fiber the patients have taken, it will present obvious benefits as soon as it enters the intestinal tract, especially, to those who are already low in dietary fiber intake (Letourneau et al., 2022). Mechanically, SCFAs activate G protein-coupled receptors, such as GPR41 and GPR43, in intestinal and adipose tissues (Lu et al., 2016). The activation of GPR41 increases glucagon-like peptide 1 and peptide YY (PYY) secretion by enteroendocrine cells, resulting in the decreased intestinal motility but enhanced nutrient absorption. GPR43 activation inhibits adipocyte differentiation, and increases hepatic lipogenesis, thereby promoting the development of NAFLD (Kimura et al., 2020).

BAs are the main components of bile and can be divided into primary and secondary bile acids. Primary bile acids (PBAs), including cholic acid (CA) and chenodeoxycholic acid (CDCA), are produced from cholesterol in hepatocytes and excreted to the bile duct. Secondary bile acids (SBAs), including lithocholic acid (LCA), deoxycholic acid (DCA), ursodeoxycholic acid (UDCA) and their respective isoforms (such as isolithocholic acid), are then transformed from the primary bile acids by bacteria in the small intestine. PBAs not only maintain intestinal microbiota homeostasis through directly inhibiting overgrowth of pathogenic bacteria, but also act as the naturally occurring agonists of intestinal FXR (FXR activation: CDCA>DCA>LCA>CA; FXR inhibition: UDCA) and subsequently activate its downstream defense genes in ileal mucosa (Fiorucci and Distrutti, 2019) to protect intestinal epithelial cells against bacterium and microorganism’s corrosion. In addition, FXR down-regulates the expression of LXR and SREBP-1c to reduce fatty acid and triglyceride synthesis in the liver, thereby reducing steatogenesis and gluconeogenesis. FXR also up-regulates hepatic glycogen synthesis *via* the activation of fibroblast growth factor (FGF) 15/19, PPARγ, GLUT-4 and GLP-1 to improve insulin sensitivity (Yang et al., 2010; Han et al., 2019). TGR5 is another classical BA receptor, mainly activated by SBAs (TGR5 activation: LCA>DCA> CDCA>CA). Too few SBAs lead to reduced FXR activity but increased inflammation in the body, while too many SBAs can cause cellular DNA damage through the production of reactive oxygen species (ROS), leading to the development of HCC. It had been found that activation of TGR5 by SBAs induces transcription of the type 2 iodothyronine deiodinase (Dio2) gene, which in turn converts thyroid hormone (T4) to the more active triiodothyronine (T3), thereby increasing basal metabolism and promoting energy metabolism in brown adiposes and muscle tissues of the high-fat diet (HFD)-fed mice (Zietak and Kozak, 2016). Activation of TGR5 in intestinal secretion cells by SBAs promotes the expression of GLP1, which increases insulin synthesis and release, resulting in the protection against islet β-cell apoptosis and improvement of blood glucose (Maczewsky et al., 2019). However, alteration of gut microbiota and hepatic function directly affects the ultimate compositions and the contents of BAs, resulting in the differential expression profiles of BA receptors (e.g., FXR vs. TGR5) among different stages of NAFLD process. Anyway, these two BA receptors, FXR and TGR5, have emerged as putative therapeutic targets for obesity and NAFLD.

Choline and its derivatives, such as TMA and TMAO, are the major metabolites of intestinal microorganisms, and their elevated levels not only contribute to atherosclerosis but also are closely related to cholesterol and triglyceride metabolism (Li et al., 2021). Clinical trials have found that reduced choline can lead to lipid deposition in the liver by reducing the synthesis and secretion of very low-density lipoproteins (VLDL) in hepatocytes, leading to the steatohepatitis. It is the consequence which could be commonly found in methionine/choline-deficient (MCD) diet-fed rodents (Cao et al., 2022; Ding et al., 2022; Zhang et al., 2022). It has been known that the food containing dietary methylamine, choline, phosphatidylcholine and carnitine will break down into various metabolites including TMA through the catabolism of trimethylamine lytic enzymes in Proteobacteria and Firmicutes (Guerrerio et al., 2012). TMA is transferred to the liver *via* the portal vein and converted by flavin-containing monooxygenases into TMAO, which promotes the accumulation of activated leukocytes into human endothelial cells, leading to endothelial dysfunction and significantly increases the risk of atherosclerosis and cardiovascular diseases (Tan et al., 2019; Shi et al., 2022).

### Gut microbiota affects intestinal and hepatic immune function

**Figure 2:**
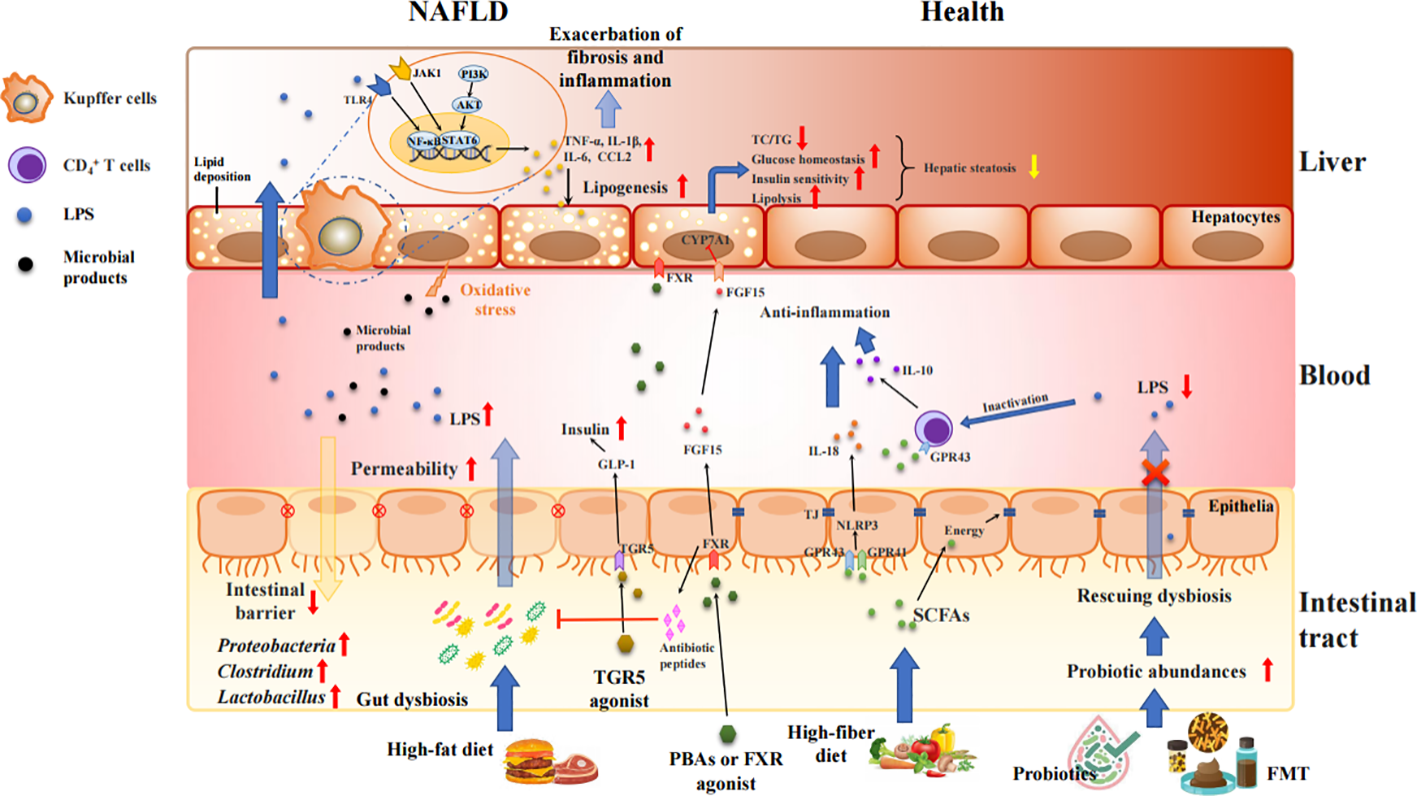
Intestinal barrier disrupted by gut dysbiosis facilitates bacterial endotoxins to liver and exacerbates inflammatory process and fat accumulation in the pathogenesis of NAFLD. Unhealth lifestyle (e.g., high-fat, low-fiber diet) alters the microbiota colonization in gut, increases gut permeability, and produces various proinflammatory molecules, such as LPS, TMAO, SBAs, and bacterial 16sDNA. These proinflammatory molecules worsen the liver inflammation and fibrosis and potentially accelerate NAFLD progression. Treatment with FXR/TGR5 agonist, probiotics and FMT strengthens intestinal tight junction, and mediates the glucose and lipid metabolism *via* activation of FXR and TGR5 signaling as well as inhibition of TLR4//NF-κB and JAK1/STAT6 pathways.

Growing investigations held the intestinal barrier dysfunction blame for inflaming the NAFLD progression (Liu et al., 2021; Gupta et al., 2022; He et al., 2022; Leng et al., 2022; Kaushal et al., 2022; Yu et al., 2022). During the initiation and progression of NAFLD, large amounts of gut bacterial metabolites, bacterial components, and other hazards enter the liver through the portal vein due to the intestinal mucosal barrier disruption induced by various stimuli, with the increased intestinal permeability. Those attacks can further accelerate liver injury and fibrosis *via* elevating inflammation, oxidative stress, and lipid accumulation (Lechner et al., 2020) as shown in **Figure 2**. The investigation by *in situ* hybridization revealed the presence of gut bacterial metabolites and DNA fragments in the livers of HFD-fed mice, but presence of bacteria in the liver of NASH patients remains unkown (Mouries et al., 2019). Compared with normal individuals, the number of enteric bacteria, especially Gram-negative bacteria, was significantly increased in obesity or NAFLD patients, and presented obvious endotoxemia (Li et al., 2022). The excessive amounts of LPS activates adenylate cyclase in the intestinal mucosa and damages the mitochondria and lysosomes of epithelial cells, leading to necrosis of the apical cells of the intestinal villi and autolysis of the epithelial cells. Moreover, the gut-derived lipopolysaccharides (LPS) are the key factor in conducting the liver inflammation and chronic damage through activating LPS-dependent pattern recognition receptor signaling (An et al., 2022). LPS activates TLR4 in endothelial cells and TLR9 in dendritic cells, resulting in the release of a large number of pro-inflammatory cytokines (such as TNF-α, IL-1β, and IL-6) and chemokines (CCL2, CXCL2, CXCL10 and CXCL16) that mediates inflammation and pathological damage to the liver (Arelaki et al., 2022; Han et al., 2022; Nagata et al., 2022; Tiegs and Horst, 2022). Therefore, LPS causes inflammation and serious metabolic changes in the body, such as increasing fat consumption, and elevating circulating free fatty acid (FFA) and triglyceride (TG). The deposition of FFA in the liver may also induce inflammation, and insulin resistance (IR), further contributing to the development of NAFLD (Rennert et al., 2020; Zheng et al., 2022).

On the other hand, the gut microbiota can alter the balance of anti-inflammatory and proinflammatory cytokines secreted by M1 and M2 macrophages by affecting the metabolism of SBAs, thereby affecting the immune function of the liver (Yang et al., 2021). Even small amounts of SBAs produced by the gut microbiota can decrease FXR activity and increase inflammation in the body; however, high amounts of SBAs can produce large amounts of ROS, cause cellular DNA damage, and lead to the development of HCC (Sun et al., 2020). In addition, PBAs can upregulate CXCL16 in hepatic vascular endothelial cells, which in turn leads to the recruitment of NKT cells that can kill tumor cells in a CD1d-dependent manner (Ma et al., 2018).

#### Gut microbiota as a screening marker and therapeutic target for NAFLD

Several studies have shown that Patients with NAFLD have the remarkable gut dysbiosis, in which the relative abundances of *Proteus* and *Enterobacter* bacteria were increased while and *Ruminococcus* and *Lactobacillus* were decreased (Loomba et al., 2019; Oh et al., 2020). As simple steatosis progresses to advanced liver fibrosis, the number of Gram-negative bacteria was increases, especially *Proteus* bacteria (Caussy et al., 2018; Pettinelli et al., 2022). Fecal BAs may reflect alterations in two bile acid metabolic pathways: “glycine, serine, and threonine metabolism” and “taurine and hypotaurine metabolism.” *Escherichia*, *Bilophila* and *Rhodobacter* involved in BAs-mediating taurine and glycine metabolism were significantly increased in the faeces of NASH patients (Jiao et al., 2018). Moreover, 60% of NAFLD patients were associated with infection with high alcohol *Klebsiella pneumoniae* (HiAlc-Kpn), and more interestingly, transplantation of this HiAlc-Kpn isolated from the feces of NASH patients could induce NAFLD in the mice (Yuan et al., 2019). However, the profiles of gut microbiota found in different studies often vary greatly due to differences in study subjects, dietary habits, medication history, and detection methods. Although close interactions between gut microbiota and NAFLD pathogenesis have been demonstrated over the past decades, the reliable microbiome markers for early NAFLD still remain to be further verified. Recently, with the aid of machine learning model integrating baseline microbial signatures to distinguish liver fat accumulation status of NAFLD participants has displayed good perspective in clinical practice, with 80% of overall accuracy (vs 60% by blood biochemical detection) (Leung et al., 2022). Given the obvious individual differences and dynamic changes of gut microbiota, it is difficult to classify disease status by stool-based biomarker detection using 16s rDNA amplification alone rather than multi-omics analysis, such as metagenomics and other culture-independent technologies. To increase the robustness and generalizability of predictors, the set of predictive metabolites identified by multiple methods could be more potential for future trials to validate prediction biomarkers in larger and independent cohorts.

## Novel strategy for NAFLD treatment based on gut microbiota

Up to now, the basic steps in the prevention of NAFLD are still diet, exercise and management of complications (such as type 2 diabetes and dyslipidemia) (Yaskolka et al., 2021; Cui et al., 2022; Cheng et al., 2022). There are few effective drugs for treatment and prevention of NAFLD/NASH. Pioglitazone may be effective in advanced NASH patients or associated with type 2 diabetes, but reliable clinical data are lacking (Shaaban et al., 2022); vitamin E and D have some efficacy, but the safety of long-term use is unclear and the course of treatment is inconclusive (Perumpail et al., 2018; Ravaioli et al., 2022); statins can reduce serum LDL levels and prevent cardiovascular complications, but do not address the progression of liver disease (Torres-Peña et al., 2021). Some promising drugs, including BA derivatives, BA metabolic-mediated agents, and probiotics, are still being evaluated in clinical trials (**Table 1**).

**Table 1:** Current clinical status of targeting gut microbiota-related agents for NAFLD treatment.

Agent	Clinical status	Target	Target diseases	Effects	References
BAs sequestrant	Approved	Increase BAs excretion	Hyperlipidemia, NAFLD	Increase hepatic LDLR and insulin sensitivity; decrease plasma LDL	(Le et al., 2012)
CDCA	Approved	FXR agonist	NAFLD	Improve liver function and reduce endogenous BAs synthesis	(Panzitt et al., 2022)
Obeticholic acid (INT-747, 6-ECDCA, OCA)	Approved	FXR agonist	NAFLD, Primary Biliary Cholangitis	Activate FXR but indirectly inhibit CYP7A1	(Younossi et al., 2019)
PX-104	Phase II	FXR agonist	Non-diabetic NAFLD	Improve insulin sensitivity and liver enzymes	(Traussnigg et al., 2021)
Nidufexor (LMB763)	Phase II	FXR agonist	NASH and diabetic nephropathy	Reduce NAFLD activity scores, triglyceride levels, and liver fibrosis	(Chianelli et al., 2020)
Cilofexor	Phase III	FXR agonist, insulin sensitizer	NASH and fibrosis	Decrease serum γ-glutamyltransferase, C4, and primary bile acids	(Patel et al., 2020)
EDP-305	Phase II	FXR agonist	NASH, cholangiopathies, renal fibrosis	Reduce ALT levels and liver fat content	(Ratziu et al., 2022)
INT-767	Phase I	TGR5/FXR dual agonist	NASH, alcoholic liver disease	Decrease hepatic steatosis, associated with reduced liver fatty acid synthase protein expression	(Iracheta-Vellve et al., 2018)
Allogenic FMT	Clinical investigation	Gut microbiota	Lean NAFLD	Decrease liver fat accumulation and improve gut dysbiosis	(Xue et al., 2022)
Allogenic FMT	Phase I	Gut microbiota	NAFLD	Not improve insulin resistance and hepatic PDFF, but reduce small intestinal permeability	(Craven et al., 2020)
Clostridium butyricum combined with Rosuvastatin	Clinical investigation	Gut microbiota and HMG-CoA reductase inhibitor	NAFLD	Increase contents of *Bacteroides thetaiotaomicron* and *Bifidobacteria*; reduce serum lipid, hepatic fibrotic indexes and pro-inflammatory cytokines	(Zhu et al., 2022)
VSL#3 (8 probiotic mixture)	Phase I	GLP-1	NAFLD	No effects on triglycerides, HOMA and ALT; decrease BMI and increase GLP-1 and activated GLP1	(Alisi et al., 2014)
Six probiotic mixtures	Phase I	Gut microbiota	Obese NAFLD	Reduce intrahepatic fat and body weight	(Ahn et al., 2019)
Synbiotics	Phase I	Gut microbiota	NAFLD	Increase proportions of Bifidobacterium and Faecalibacterium, and decrease Oscillibacter and Alistipes; not reduce liver fat content or markers of liver fibrosis.	(Scorletti et al., 2020)
Probiotics	Clinical investigation	Gut barrier	NAFLD	No significant clinical improvement	(Mohamad et al., 2021)
Probiotics	Clinical investigation	Insulin resistance	NAFLD	Decrease blood glucose, insulin, insulin resistance, TNF-α, and IL-6	(Sepideh et al., 2016)

### Microbiome-targeted therapies

MTT is a novel strategy for the treatment of NAFLD by correcting or reshaping gut microbiota by using antibiotics, probiotics, prebiotics, synbiotics and FMT to maintain gut homeostasis (Sharpton et al., 2019). Commercialized *Streptococcus*, *Lactobacillus*, and *Bifidobacterium* improve the gut inflammatory microenvironment, promote the growth and survival of intestinal epithelial cells, and inhibit pathogenic bacteria by modulating the immune system and host defense (Xie et al., 2018). As safe edible probiotics, *Lactobacillus* and *Bifidobacterium* can lower blood cholesterol levels (Wu and Chiou, 2021). FMT is a comprehensive approach for regulating the gut microbiota that has been shown to be effective in restoring microbiota composition and can be used to treat *C. difficile*-associated diarrhea and *Pseudomembranous colitis*, as well as other chronic intestinal inflammatory diseases, such as ulcerative colitis and Crohn’s disease (Costello et al., 2019; Sokol et al., 2020). After FMT treatment, the gut microbiota in both HFD-fed mice and NAFLD patients would be reshaped, small intestinal permeability was elevated, and the symptom of NASH was partly improved (Zhou et al., 2017; Craven et al., 2020; Xue et al., 2022). However, unlike the necessity for ulcerative colitis and Crohn’s disease, FMT is not often the first choice for NAFLD patients due to the distaste for the gut contents. Compared with FMT, prebiotic supplementation has a higher safety and operability. The oral administration of prebiotics, such as lactulose, oligofructose, and inulin, can stimulate the release of gastrointestinal peptides, which have the effect of regulating appetite and energy metabolism of the body (Cerdó et al., 2019). However, the excessive intake of inulin may cause liver damage, and thus prebiotics should be supplemented appropriately under the guidance of a doctor to prevent harm to the body (Singh et al., 2018). Although probiotics, prebiotics and synbiotics have been proposed for treating the patients with obesity-related NAFLD and indeed alleviate intestinal permeability, they did not show any significant clinical improvement in NAFLD patients (Behrouz et al., 2020; Scorletti et al., 2020; Chong et al., 2021; Mohamad et al., 2021). Therefore, their therapeutic effects should be further investigated by high-quality clinical trials.

### Selective intervention of specific flora (phage therapy)

The use of a specific bacteria or bacterial products to interfere with NASH may be a novel therapeutic strategy for anti-NAFLD drug discovery. The colonization of *Akkermansia muciniphila* in gut could improve liver function, reduce oxidative stress, inhibit inflammation, normalize gut microbiota, and reverse metabolic disturbances caused by HFD (Jiao et al., 2018). Some SBA-producing bacteria, such as *Lactobacillaceae* and *Lachnospiraceae*, exhibit cholesterol-lowering potential and may improve metabolic abnormalities under HFD (Zeng et al., 2020).

Recently, phage therapy is being given great attention as a new antimicrobial agent. Understanding endogenous phage-gut microbiota interactions in health and in NAFLD may enable phage utilization in precise gut microbiome editing, towards treating NAFLD and other obesity-associated metabolic diseases. As previously reported, the targeted eradication of HiAlc-Kpn with phages effectively alleviates bacterial auto-wine syndrome and NASH in NAFLD model mice (Yuan et al., 2019). Treatment with bacteriophages can specifically target cytolytic *Enterococcus faecalis* and alleviate liver injury in humanized mice colonized with faecal bacteria of patients with alcoholic hepatitis (Duan et al., 2019). Unlike the major therapeutic hurdles of antibiotics and other chemotherapies caused by high degrees of bacteria resistance in clinic, phage therapy provides a new therapeutic approach for precisely editing the intestinal microbiota, completely resolving bacteria resistance and treating liver diseases. Although great advances have presented in various bacterial infection (Mitropoulou et al., 2022; Van Nieuwenhuyse et al., 2022), due to the diversity and complexity of gut microbiome, more clinical trials with larger cohorts are required to validate the relevance of the findings in NAFLD patients.

### The interaction between gut microbiota and immunotherapy in NAFLD

The gut microbiota also has been demonstrated to enhance the therapeutic response to immune checkpoint inhibitors though targeting immunomodulatory molecules or their ligands on the surfaces of T cells (McCulloch et al., 2022; Wu et al., 2022; Zhang et al., 2022; Zhao et al., 2022). The combination has been approved for treating various malignant tumors, including NAFLD-associated HCC (Baruch et al., 2021; Davar et al., 2021; Shiraishi et al., 2022). Compared with those untreated with antibiotics, progression-free survival and overall survival of patients treated with antibiotics were significantly lower during the treatment of anti-PD-1/PD-L1 antibodies (He et al., 2021). This finding was consistent with the results of the mouse experiment, in which the transplantation of fecal flora from tumor patients who responded to PD-1 inhibitor into germ-free mice enhanced the antitumor effects of PD-1 inhibitor, while Fecal flora from non-responding patients did not exhibit this improvement (Gao et al., 2021). These results suggest that the regulation of gut microbiota may be a new target for NAFLD-related HCC and cirrhosis therapy. Given that patients with HCC and cirrhosis often have severe intestinal ecological dysregulation, which may lead to immunotherapy failure in some patients, the modulation of the gut microbiota may be more relevant for the therapeutic response in HCC and cirrhosis than other solid tumors.

Notably, recent data indicate that the IL-17/IL-17R originating from TH17 cells drives intestinal neutrophil migration, limits gut dysbiosis, maintains intestinal barrier integrity and attenuates LPS translocation to visceral adipose tissue, resulting in protection to metabolic syndrome and comorbidities such as NAFLD and obesity (Pérez et al., 2019; He et al., 2022). Paradoxically, there is also some evidence that IL-17 accelerated hepatic steatosis through activation of the JNK-PPARα pathway in the HFD mice and oleic acid-preloaded hepatocytes (Shen et al., 2017; Sugimura et al., 2019; Pan et al., 2019; Wang et al., 2020; Wang et al., 2021; Takamura et al., 2022). Circulating levels of IL-17, released by the visceral adipose tissue, induces eotaxin secretion *via* the smooth muscle cells, both of which are associated with early atherosclerosis that is the main factor drastically reducing the survival of NAFLD patients (Tarantino et al., 2014). These results indicated the versatile of IL-17 in the development of NAFLD and the significance of target delivery in different tissues.

### FXR and TGR5 agonists

Besides the central roles in bile acid metabolism, FXR and TGR5 are closely linked to the metabolism of lipids, glucose and lipoproteins, and hence have emerged as the new targets for the treatment of NAFLD (Yang et al., 2010; Han et al., 2019; Maczewsky et al., 2019). FXR agonists include natural ligands (such as CDCA), semi-synthetic BAs (such as obeticholic acid (OCA)) or synthetic non-steroidal molecules (such as GW4064, WAY-362450). These FXR agonists prevent hepatic steatosis in obese and insulin-resistant rodents. Synthetic FXR agonists are associated with increased body mass and reduced glucose tolerance with prolonged use, whereas the BA class of natural FXR agonists are not. There are two published clinical trials on the FXR agonist OCA. Short-term oral administration of OCA improved insulin resistance and reduced markers of liver fibrosis in patients with NASH combined with type 2 diabetes (Mudaliar et al., 2013; Pockros et al., 2019). However, in the FLINT (clinical trials gov identifier NCT01265498) trial, long-term oral administration of OCA at 25 mg/d for 72 weeks in patients with NASH did not improve insulin sensitivity and increased homeostasis model assessment (HOMA) indices (Neuschwander-Tetri et al., 2015). The difference between these two trials may lie in the length of time the medication is taken. Indeed, OCA treatment significantly improved liver histological outcomes, with a reduction in NASH activity scores compared with placebo-treated patients. However, the improvement rate was only 22% and hardly seen in patients with severe fibrosis (Younossi et al., 2019). Also, the adverse reactions caused by FXR agonist are the key problems that have to be faced. Therefore, further clinical trials are needed to assess the efficacy and safety of OCA for the clinical treatment of NASH.

Up to now, most synthetic FXR agonists are ongoing at preclinical or phase I status (Panzitt et al., 2022). Recent study has shown that hepatic FXR subtypes (α1 and α2) differ in their mechanisms of limiting lipid accumulation in hepatocytes, in which FXRα2 has a stronger inhibition on hepatic TG levels (Ramos et al., 2020). This has implications for the further optimization of FXR agonists and the improvement of therapeutic efficacy.

TGR5 agonists are theoretically able to maintain glucose homeostasis, increase energy consumption to maintain healthy body mass, and reduce liver inflammation in NASH. The CA derivative INT-777 is a selective TGR5 agonist that increases energy metabolism, reduces liver fat deposition and prevents body mass gain in HFD-fed mice (de Oliveira et al., 2016; Gillard et al., 2022). The TGR5/FXR dual agonist INT-767 showed good affinity to both receptors, and reduced hepatic steatosis in obese diabetic mice *via* repressing the production of pro-inflammatory cytokines and inducing M2 macrophage polarization (McMahan et al., 2013; Iracheta-Vellve et al., 2018). Despite good results in animal studies, clinical trials with TGR5 agonists have been less effective.

UDCA is a classical drug with immunomodulatory, anti-oxidant and anti-apoptotic effects. However, its effect on NAFLD/NASH has not been confirmed in clinical trials (Marchianò et al., 2022). However, some derivatives of UDCA have shown good therapeutic effects in mouse NASH models, such as norUDCA, which is currently in phase II clinical trials.

The BA sequestrants colesevelam interrupts the hepatic-intestinal circulation of BA and has been shown to affect cholesterol, triglyceride and glucose homeostasis, reducing hepatic fat deposition in NASH patients (Kessoku et al., 2020). Several additional ASBT antagonists have been developed for the treatment of hyperlipidemia and hypercholesterolemia, but have not been tested in NAFLD (Ge et al., 2019; Karpen et al., 2020).

## Future challenges and prospects

Abnormal gut microbiota can promote the occurrence, progression, and even deterioration and carcinogenesis of NAFLD. How to use gut microbiota to alter or block the progression of NASH and find bacteria and bacterial products, as potential targets, has become the critical for NAFLD intervention and treatment. First, although new strategies, such as probiotics, prebiotics, FMT, phage therapy and FXR/TGR5 agonists, have shown promise in the treatment of NAFLD, challenges in developing safe and effective therapeutic drugs, including the selectivity, tissue specificity and drug resistance for long-term use, remain further assessed.

Second, whether specific bacterial strain or microbiota are causally related to the occurrence of NASH and could be served as effective therapeutics has not yet been established mechanistically. Moreover, most studies have remained at the animal stage, and how differences in gut bacterial species between animals and humans affect microbial metabolism remains unclear.

Third, other microbial factors, such as metabolites associated with gut virus, may be also important to the progression of NAFLD. To date, the microbial origins of many detected metabolites are unclear, and few microbial sources have been reported for several metabolites. The metabolomic profiles of patients with NAFLD are inconsistent and sometimes contradictory probably because of the relatively small number of patients and the lack of uniform methods and standards. Therefore, in-depth studies are required to explore these metabolites and their microbial sources and to refine the characteristics of floras and their metabolites in different NAFLD subtypes. It is believed that the developments in technology and research may facilitate the use of the gut microbiota in NAFLD intervention or treatment, and the in-depth exploration of the composition and function of gut microbiota will bring new diagnostic tools and personalized treatments for NAFLD.

## Author contributions

JF, and C-HY: Wrote the manuscript; X-JL, J-MY, S-HY, and Z-YF: Searched literatures; C-HY, and JF: Illustrated figures and tables; Z-YF, and W-YY: Edited the manuscript; C-HY and W-YY: Designed and supervised the review. All authors contributed to the article and approved the submitted version.

## Funding

This work was supported by Natural Science Foundation of Zhejiang Province (No. LZ17H280001 and LY19H200003), Key research projects of Hangzhou Medical College (No. KYZD202106), and Key Laboratory of Neuropsychiatric Drug Research of Zhejiang Province (No.2019E10021).

## Conflict of interest

The authors declare that the research was conducted in the absence of any commercial or financial relationships that could be construed as a potential conflict of interest.

## Publisher’s note

All claims expressed in this article are solely those of the authors and do not necessarily represent those of their affiliated organizations, or those of the publisher, the editors and the reviewers. Any product that may be evaluated in this article, or claim that may be made by its manufacturer, is not guaranteed or endorsed by the publisher.
